# Therapeutic Approach of Flavonoid in Ameliorating Diabetic Cardiomyopathy by Targeting Mitochondrial-Induced Oxidative Stress

**DOI:** 10.3390/ijms222111616

**Published:** 2021-10-27

**Authors:** Syaifuzah Sapian, Izatus Shima Taib, Jalifah Latip, Haliza Katas, Kok-Yong Chin, Nor Anizah Mohd Nor, Fatin Farhana Jubaidi, Siti Balkis Budin

**Affiliations:** 1 Centre for Diagnostic, Therapeutic and Investigative Studies, Faculty of Health Sciences, Universiti Kebangsaan Malaysia, Kuala Lumpur 50300, Malaysia; syaifuzahsapian17@gmail.com (S.S.); izatusshima@ukm.edu.my (I.S.T.); ejamdnor@gmail.com (N.A.M.N.); fatinfarhanajubaidi@gmail.com (F.F.J.); 2 School of Chemical Sciences and Food Technology, Faculty of Science and Technology, Universiti Kebangsaan Malaysia, Bangi 46300, Malaysia; jalifah@ukm.edu.my; 3 Centre for Drug Delivery Research, Faculty of Pharmacy, Universiti Kebangsaan Malaysia, Kuala Lumpur 50300, Malaysia; haliza.katas@ukm.edu.my; 4 Department of Pharmacology, Universiti Kebangsaan Malaysia Medical Centre, Kuala Lumpur 56000, Malaysia; chinkokyong@ppukm.ukm.edu.my

**Keywords:** flavone, flavanone, flavonol, flavanol, isoflavone, anthocyanin, reactive oxygen species

## Abstract

Diabetes cardiomyopathy is one of the key factors of mortality among diabetic patients around the globe. One of the prior contributors to the progression of diabetic cardiomyopathy is cardiac mitochondrial dysfunction. The cardiac mitochondrial dysfunction can induce oxidative stress in cardiomyocytes and was found to be the cause of majority of the heart morphological and dynamical changes in diabetic cardiomyopathy. To slow down the occurrence of diabetic cardiomyopathy, it is crucial to discover therapeutic agents that target mitochondrial-induced oxidative stress. Flavonoid is a plentiful phytochemical in plants that shows a wide range of biological actions against human diseases. Flavonoids have been extensively documented for their ability to protect the heart from diabetic cardiomyopathy. Flavonoids’ ability to alleviate diabetic cardiomyopathy is primarily attributed to their antioxidant properties. In this review, we present the mechanisms involved in flavonoid therapies in ameliorating mitochondrial-induced oxidative stress in diabetic cardiomyopathy.

## 1. Introduction

Diabetes mellitus (DM) is one of the most deadly non-communicable diseases that leads to extensive impairments of organs and body functions. The increasing incidence of DM and its related complications have contributed to the surge of morbidity and mortality rate. DM affects about 463 million people aged between 20 to 79 years in 2019, and this figure is expected to climb up to 700 million by 2045 [1]. Moreover, DM is also one of the root causes for the development of cardiovascular diseases (CVD), which further exacerbate the mortality risk among patients with DM [2]. One of the complications resulting from chronic DM is diabetic cardiomyopathy (DCM). DCM is a cardiac pathological condition in patients with DM characterized by the appearance of aberrant myocardial morphology and cardiac functions in the truancy of other factors, such as coronary artery disease, hypertension, and prominent valvular disease [3]. Due to DCM, patients with DM are more likely to suffer from heart failure compared to their healthy counterparts [3]. This is why DCM is one of the most devastating consequences directly caused by DM.

The heart has a high energy consumption in order to efficiently pump and supply blood throughout the body. Hence, it has a high density of mitochondria population to fuel its activities. However, this dependence exposes the heart to deleterious consequences when mitochondrial malfunction occurs. Mitochondria serve a critical part in oxygen metabolism, hence it is crucial to understand the effects of their dysfunction in patients suffering from metabolic disorders, particularly diabetes [4]. In DCM, the minimal glucose utilization will shift to fatty acid, leading to energetic inefficiency [5]. Since the mitochondria lost its efficiency in energy production, mitochondrial dysfunction will then follow. The role of mitochondrial dysfunction in the progression of DCM has been well established in earlier studies [6,7,8]. As the heart contains a high amount of mitochondria, cardiac mitochondrial dysfunction can lead to cardiac oxidative stress which aggravates the development of DCM. Indeed, the diabetic patients heart mitochondria are typically found to have deteriorated in number and structure, exhibiting increased reactive oxygen species (ROS) emission, and compromised mitochondrial respiratory capacity in the mitochondria [9]. Thus, treatment targeting mitochondrial-induced oxidative stress is very crucial in suppressing DCM.

For more than 40 years, the pathogenesis and mechanisms involved in DCM’s development and progression has been well-studied and documented as well as of its preventive measures and potential therapeutic agents. Despite this, effective remedies for preventing and treating DCM remains unclear [10]. The need of having a treatment for DCM is of utmost importance considering that there is no specific treatment targeting DCM up to the moment [11,12]. Presently, diabetes management is only based on combination of lifestyle modification and therapeutic medications to regulate blood glucose level through glucose-lowering agents or insulin replacement therapy as well as with management of cardiovascular complications [12,13].

Recently, extensive efforts have been invested in studying the use of natural compounds to treat DCM. One of the candidates is flavonoids, which are plant-based polyphenolic compounds found in abundance in some fruits, vegetables, and herbal plants. They have been reported to exert many therapeutic effects against various pathologic conditions, such as cancer, muscle atrophy, inflammation, microbial infection, oxidative stress as well as DM [14,15,16,17,18,19]. These therapeutic effects are mainly mediated through radical scavenging, antioxidant, and anti-inflammatory properties [20,21,22]. Furthermore, flavonoids have gained recognition for their cardioprotective capabilities. Flavonoids have been proven to attenuate the progression of DCM via mitochondrial protection, thereby shielding cardiomyocytes against mitochondrial-induced oxidative stress [23,24,25]. Since flavonoids had showed potentials in alleviating cardiac dysfunction, we sought to review the therapeutic approach of flavonoids in ameliorating diabetic cardiomyopathy by targeting mitochondrial-induced oxidative stress.

## 2. Diabetic Cardiomyopathy

Rubler et al. [26] was the first to propose the concept of DCM, which has since become widely used in medicine. For decades, there has been an epidemiological relationship found between DM and the pathophysiology of heart failure. The prevalence of DCM is increasing simultaneously with the increased incidence of DM, and it is a main contributing factor to the pathophysiology of heart failure in DM patients [27]. DCM has a long latent phase during which the disease develops silently without observable symptoms. Upon comprehensive clinical investigation, patients may display increased in ventricular mass, substantial myocardial fibrosis, impaired cardiac cell signaling, and diastolic dysfunction which all are features of the early stage of DCM [3,5]. Patients with DCM typically start to exhibit symptoms as systolic function was exacerbated by diabetes-induced uncontrolled cardiac remodeling, which is usually permanent and irreversible, thus progressing towards heart failure [28,29].

Both type 1 (insulin dependent) and type 2 (non-insulin dependent) DM share the same feature, which is hyperglycemia that results from poor insulin action. Chronic hyperglycemia is indeed one of the major components that exacerbate the progression of cardiovascular complications in DM patients. In fact, a study showed that incidence of heart failure among DM patients rise by 8% with every 1% increase in glycated hemoglobin (HbA1c) level [30]. Oxidative stress is a contributing factor for DCM progression [31]. Uncontrolled and persistent hyperglycemia encourage excessive generation of reactive oxygen species (ROS) via several metabolic pathways; elevated glucose uptake through the polyol pathway, enhanced advanced glycation end products (AGEs) production, activation of protein kinase C (PKC) pathway, overactivation of hexosamine pathway and incapacitated antioxidant defense [32].

**Figure 1:**
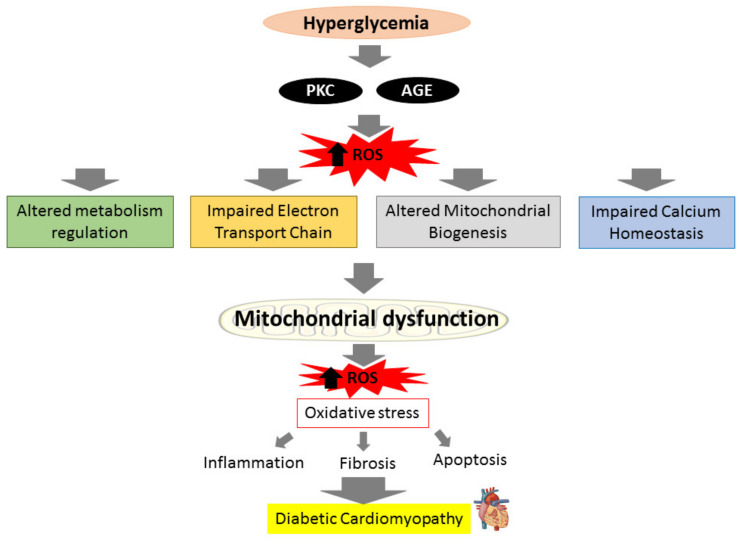
Prolonged hyperglycemia can produce reactive oxygen species (ROS) via activation of protein kinase C (PKC) pathways and advanced glycation end products (AGEs) production, leading to altered metabolism regulation, altered mitochondrial biogenesis, impaired mitochondrial calcium handling, and impaired electron transport chain. These actions will cause mitochondrial to deteriorate and generate more ROS. The ROS produced results in oxidative stress, which can initiate inflammation, fibrosis, and apoptosis, causing diabetic cardiomyopathy (DCM).

The heart’s energy demands are constantly high due to its need to maintain specialized cellular functions, and cardiomyocytes mitochondria generate more than 95% of their adenine triphosphate (ATP) by oxidative phosphorylation (OXPHOS) [33]. In DM condition, the insulin impairment and inability to utilize glucose in mitochondria will switch from glucose to fatty acid oxidation (FAO) to produce ATP in order to maintain sufficient ATP generation. This process however produces more ROS and causes the OXPHOS process to be disrupted [34]. The cytosolic ROS produced can promote mitochondrial dysfunction by attacking mitochondrial structure further. Upon breach in their structure, fragments of mitochondrial deoxyribonucleic acid (DNA) are released into the cytosol and triggers cardiac inflammation and stimulate the release of pro-inflammatory cytokines which exacerbate the inflammatory process that lead to more mitochondrial damage and loss of function [35,36]. Impaired mitochondria create more ROS, resulting in even worse oxidative damage [37]. ROS accumulation from both mitochondrial dysfunction and hyperglycemia-induced activation of metabolic pathways augments myocardial oxidative stress and aggravates mitochondrial dysfunction, resulting in cardiomyocyte death and the subsequent remodeling of the heart structure in effort to preserve the heart integrity and function. The heart’s ability to contract and relax efficiently is further harmed by uncontrolled and excessive cardiac fibrosis and hypertrophy that lead to irreversible cardiac structural changes, ultimately results in diastolic and systolic dysfunction in DCM [29,38]. The relationship between hyperglycemia and mitochondrial dysfunction towards the progression of DCM is illustrated in brief in Figure 1.

## 3. Mitochondrial-Induced Oxidative Stress in Diabetic Cardiomyopathy

Mitochondrial dysfunction is the destruction of mitochondrial morphology, respiratory chain disruption, biogenic dysfunction, gene alterations, mitochondrial population deprivation, and alteration in the oxidative protein’s activity in cells [39]. Although ROS is the main by-product of oxygen metabolism by the mitochondria, hyperglycemic condition may also induce its excessive production. This results in the accumulation of the ROS, leading to mitochondrial oxidative damage that attack its protein, DNA, and lipid structures [40]. As mitochondrial DNA are lacking histone protection, it is very susceptible to oxidative damage which disrupts its respiratory chains and biogenesis [41]. Accumulation of ROS not only will disrupt the mitochondrial normal functions, but also induces the development of mitochondrial permeability transition pores (mPTP) that leads to the depolarization of the mitochondrial membrane and release of factors of cell death into the cytosol [42].

### 3.1. Altered Metabolic Regulation

Cardiomyocytes are high-energy-consuming cells with mitochondria as their primary source of energy supply. Mitochondria is a major organelle for glucose and fatty acid metabolism. Impaired mitochondrial activity can impede insulin signaling by meddling with acyl-CoA oxidation from fatty acid, diacylglycerol accumulation, PKC stimulation, AGEs production and ROS formation [43]. Under normal circumstances, β-oxidation accounts for around 70% of the energy supply in the heart, with the rest coming from the oxidation of other substances such as glucose, ketone bodies, lactate, and amino acids. [44]. It is worth bringing up that fatty acids, as energy metabolic sources, require around 12% more oxygen to generate the same proportion of ATP as glucose. Nevertheless, FAO increases while glucose oxidation decreases. In DCM patients, FAO is the primary source of ATP generation, which can result in increased oxygen demand and respiratory dysfunction in mitochondria. [45].

In DCM condition, the surge in serum fatty acid promotes an increase in fatty acid utilization and FAO. Hyperglycemic condition downregulate activation of 5′ adenosine monophosphate-activated protein kinase (AMPK) and causes reduction in peroxisome proliferator-activated receptor-gamma coactivator 1 (PGC-1) regulation. The FAO rate increases in conjunction with the decreased peroxisome proliferator-activated receptors (PPARs) activity, including PPARα activity where its activation is triggered by PGC-1. However, acyl-CoA overload generated from excess fatty acid can lead to excessive mitochondrial ROS generation [46]. Particularly, the production of byproduct from β-oxidation such as nicotinamide adenine dinucleotide (NAD) + hydrogen (H) (NADH) and flavin adenine dinucleotide (FADH_2_) are both increased in excess, leading to generation of ROS in the electron transport chain (ETC) [47]. Downregulation of the cardiac-specific manganese superoxide dismutase (MnSOD) or AMPK activity further elevates ROS production in the mitochondria, which allows mitochondrial disruptions and FAO [8,48].

Intriguingly, elevated free fatty acid uptake has been linked with the surge of uncoupling protein 3 (UCP3) in cardiac muscle, whose function is to facilitate anion transfer from inner to outer membrane of the mitochondria [49,50]. In cardiomyocytes, UCP3 is upregulated by increase circulating free fatty acids via activation of PPARα activation [51]. Even though UCP3 appears to specifically involve in encouraging fatty acid oxidation, it is indirectly influencing glucose homeostasis [52]. Subsequently, UCP3 reduced mitochondrial electrochemical gradient which further deprived ATP generation [53]. In addition, proton leak from the OXPHOS that triggered by increase of FAO also enhance the regulation of UCP3 as proton leakage is precisely regulated and be catalyzed or suppressed by UCP3 [54].

### 3.2. Impaired Electron Transport Chain (ETC)

There is tremendous data that mitochondrial ROS generation triggers the development of DCM. Indeed, patients with DM possess defective cardiac mitochondria, with increased hydrogen peroxide outflow, reduced respiratory capability, and elevated levels of oxidized proteins [9]. One of the primary machineries that produce excessive ROS under hyperglycemic condition is the ETC itself. ETC is the primary site of mitochondrial ATP generation within all cells particularly cardiomyocytes. The ETC is made up of protein complexes I, II, III, and IV as well as the electron transfer carriers, ubiquinone (Co-enzyme) and cytochrome C, and is where ATPs are being produced during OXPHOS. At the inner mitochondrial membrane (IMM), electrons from NADH and FADH_2_, the byproduct of β-oxidation, are transferred through the respiratory chain to oxygen, which is then reduced to water at complex IV [55]. The flow of protons into the intermembrane gap is fueled by this mechanism, which creates a proton gradient which generate mitochondrial membrane potential (ΔΨm) that promotes ATP production by the ATP synthase [56]. Due to the incomplete reduction of oxygen, some electrons (approximately 0.1%) that escape from ETC can induce superoxide (ROS) generation [57].

Past studies suggested superoxide generation from ETC as the primary event in hyperglycemia-induced mitochondrial dysfunction [58,59]. High glucose levels in the cell and glucose-depleted pyruvate boost respiration by raising the ETC’s capacity, leading to mitochondrial membrane hyperpolarization and superoxide generation [60]. Superoxide formation can occur when the electron flow is reduced, especially at the first three complexes, where flavins or quinones might operate as single electron donors [55]. The generation of ROS can also be triggered by the reverse electron flow via complex I [61]. Interestingly, the protection exerted when complex I or Il were inhibited implies that ETC superoxide generation occurs via reverse electron transfer during high glucose exposure. Furthermore, many treatments, such as inhibiting ETC complex II activity, uncoupling OXPHOS, upregulation of uncoupling protein-1, or MnSOD, can reduce hyperglycemia-induced ROS production [59].

Abnormalities in hyperglycemic condition caused by oxidation can escalate methylglyoxal adduct production and elevate O-linked β-N-acetylglucosaminylation (O-GlcNAcylation). These are examples of post-translational changes that lead to mitochondrial and systolic function deterioration [62]. Hyperglycemia disrupts the activity of the respiratory mechanisms in cardiac mitochondria and causes O-GlcNAcylation dysregulation [63]. In normal circumstances, the O-GlcNAc transferase (OGT) is found in the IMM and interacts with complex IV of ETC. This enzyme is poorly localized to the mitochondrial matrix in hyperglycemic condition, and thus the OGT-complex IV connection is disrupted, resulting in lowered complex IV activity and reduced ΔΨm [63]. O-GlcNAcylation of mitochondrial dynamics proteins, including mitochondrial dynamin-related protein 1 and optic atrophy gene 1 leads to mitochondrial rupture, perpetuating mitochondrial failure [64,65].

In DCM, ATP synthase activity is typically found to be impaired, which compromises mitochondrial function. Persistent hyperglycemia induces overexpression of mitochondrial calpain-1, a calcium-activated intracellular proteinase [66]. Calpain-1 was found to cleave ATP synthase, leading to reduced ATP synthase function which triggers excessive mitochondrial superoxide formation [67]. Its activation is thought to be mediated by nicotinamide adenine dinucleotide phosphate oxidase (NOX) subunit, gp91phox [68]. These findings highlight the relevance of ETC in mitochondria as a significant source in generating superoxide in DCM.

**Figure 2:**
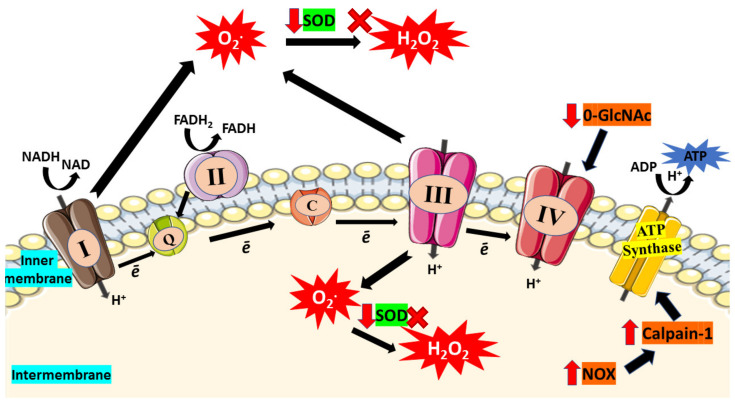
In diabetic condition, hyperglycemia causes impairment of electron transport chain (ETC). Due to incomplete reduction of oxygen, electron (ē) will escape from the ETC and lead to superoxide (O_2_*) production. Furthermore, glucose-depletion in the mitochondria boost respiration and enhance ETC capacity which then enhances production of superoxide that leads to increased consumption of SOD. Reduction in SOD activity results in the accumulation of superoxides and reduced their conversion to hydrogen peroxide (H_2_O_2_). Moreover, elevated O-GlcNAc was found in hyperglycemic condition which then reduces the activity of complex IV. The activity of ATP synthase also declines as high glucose triggers NOX expression and further enhances expression of calpain-1, leading to ATP synthase cleavage and thus reduces the production of ATP. Red arrow indicates increase/decrease of level/activity; black arrow indicates flow of mechanisms in ETC; ‘x’ symbol indicates inhibition of H_2_O_2_ production.

Apart from that, cardiolipin, a phospholipid in IMM, has been suggested to play a significant role in controlling energy generation by optimizing the IMM proteins and complexes activities involved in OXPHOS [69,70,71]. Hence, deterioration of cardiolipin structure can affect the ATP production in ETC. According to a study, streptozotocin-induced diabetic rodents displayed prominent changes in the interfibrillar of mitochondrial population, including depleted cardiolipin concentration and electron flow capacity [72]. This finding shows that depletion of cardiolipin leads to diminished electron flow capacity and consequently enhanced superoxide production from ETC. Besides, increased level of ROS can alter mitochondrial cardiolipin and leads to mitochondrial architecture disruption, including mitochondrial disintegration, cristae disruption, and swelling which have been observed in cardiomyocytes from diabetic hearts [73,74]. Figure 2 illustrated impaired ETC which then enhance oxidative stress in mitochondria.

### 3.3. Altered Mitochondrial Biogenesis

Mitochondrial biogenesis is a process by which the mitochondrial population in a cell multiplies. One of the factors that stimulate to the alterations in mitochondrial biogenesis, respiratory function, and/or lowered ATP production is diabetes. Hence, diabetes-induced impaired mitochondrial biogenesis will diminish mitochondrial function. In normal physiology, mitochondrial DNA transcription is triggered by AMPK and activated by the family PGC-1 proteins, where it is considered as the master regulator in mitochondrial biogenesis [75]. In contrast, insulin resistant uncoupling protein-diphtheria toxin a (UCP-DTA) transgenic mice showed elevation in PGC-1 expression consistent with the promotion of PPARα in the heart, whose function is known to activate metabolic genes in the heart [76]. This further confirms that PGC-1 plays a key role in mitochondrial biogenesis and that its reduced activity in diabetes condition suppresses mitochondrial biogenesis. In DCM, preliminary studies exhibited that hypoadiponectinemia impaired AMPK-PGC-1α signaling [77], more recently, in a model for type 2 DM with high fat diet, adiponectin was found to partial rescue mitochondrial biogenesis in cardiac cells, via PGC-1α-mediated signaling [78]. When mitochondrial biogenesis is disrupted, mitochondrial biogenesis is inhibited, hence ATP generation is hampered. As a result, mitochondrial ATP synthesis will rise, increasing the burden in the mitochondria, resulting in the production of ROS and oxidative damage.

### 3.4. Impaired Mitochondrial Calcium Homeostasis

One of the primary drivers for mitochondrial-induced oxidative stress has been identified to be impairment in mitochondrial calcium handling. Calcium homeostasis is important in the regulation of cellular metabolism, muscle contraction, and signal transduction [3]. Furthermore, mitochondria is an important organelle for calcium regulation and storage [79,80]. For ATP generation in mitochondria, a transmembrane potential gradient, also known as a proton (Ca^2+^) gradient, is required. A uniporter transports calcium down the concentration gradient, while a Na^+^/Ca^2+^ exchanger removes the accumulated calcium. Calcium uptake into a mitochondrion is necessary for Krebs cycle activation and ATP generation. Furthermore, calcium transport from the cytosol to the mitochondria is also responsible for regulating ATP supply and demand for cardiac function [81]. Calcium uptake in mitochondria may also function as a buffering system, eliminating local calcium and adjusting accumulated cytosolic calcium level, hence controlling the activity of calcium-dependent mitochondrial enzyme activities [82].

**Figure 3:**
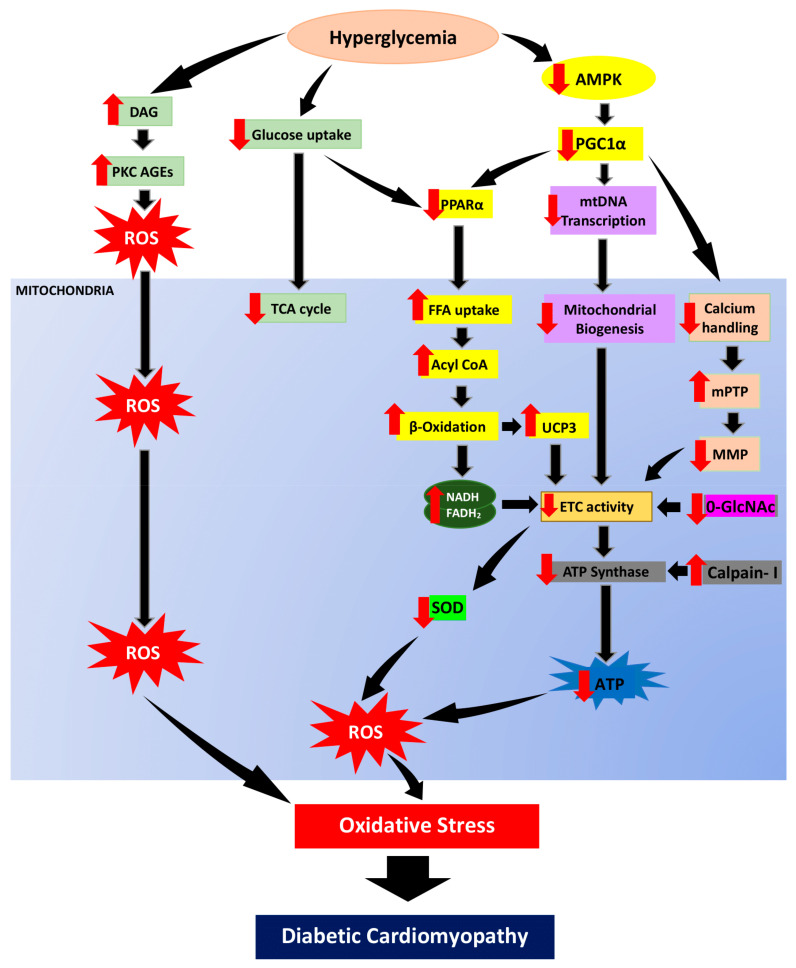
In hyperglycemic condition, reduction of glucose uptake will suppress glucose oxidation. Hence, the energy metabolism will shift from glucose to fatty acid utilization. AMPK, regulator of energy homeostasis, will be downregulated hence suppressing PGC1α expression. PGC1α suppression downregulates PPARα and enhances free fatty acid uptake, acyl CoA as well as escalating β-oxidation. In parallel with that, the TCA cycle is also deprived. The increase of DAG resulting from persistent hyperglycemia enhances PKC activation and AGEs formation which then promote ROS generation. In addition, the surge of β-oxidation produce byproduct, NADH and FADH_2_ as well as enhanced UCP3 expression further cause reduction in electron transport chain activity. Furthermore, PGC1α suppression also leads to mitochondrial biogenesis impairment via reduction of mitochondrial transcription. PGC1α suppression will also cause poor calcium handling which then enhance mitochondrial permeability transition pore (mPTP) opening and diminishes mitochondrial membrane polarization. Enhanced β-oxidation, impaired of mitochondrial biogenesis and poor calcium handling will then cause reduction of ETC hence cause overproduction of superoxide and hydrogen peroxide as well as downregulation of O-GlcNAc. Calpain-1 activity enhancement cleaves and diminish ATP synthase activity which will cause reduction in ATP production. These mechanisms of mitochondrial dysfunction are the root to oxidative stress and consequently lead to diabetic cardiomyopathy development. Black arrow indicates flow of mechanisms involved; red arrow indicates increase/decrease of level/activity.

In hyperglycemic condition, excessive calcium loading in mitochondrial matrix can result in the opening of the mPTP complex with large amounts of Ca^2+^ released into the cytoplasm, ultimately leading to activation of apoptotic factors in cardiomyocytes [83,84]. mPTP is a known cause for mitochondrial swelling, equilibration of ionic gradient, and depletion of ΔΨm, and thus leading to the impairment of ATP production in ETC. Apart from that, cardiolipin in IMM is also vulnerable to free radical and has been demonstrated to play a key role in calcium maneuver and apoptosis [85]. In addition, PGC-1 is known to be involved in mitochondrial production and respiratory activity regulated by calcium-dependent mechanisms [86]. It has been shown to have a role in calcium signaling and calcium-mediated oxidative damage [87]. Therefore, it is plausible that impaired mitochondrial calcium handling adds to oxidative stress and disrupt energy homeostasis in DCM. The overall mechanisms of mitochondrial-induced oxidative stress in DCM were illustrated in Figure 3.

## 4. Therapeutic Role of Flavonoid in Alleviating Mitochondrial Dysfunction-Induced Oxidative Stress in Diabetic Cardiomyopathy

Flavonoids are one of the most diverse families of bioactive phytochemicals, with over 9000 different compounds. According to IUPAC Recommendations (2017), the term “flavonoid” refers to compounds that have the basic structure of phenyl-substituted propylbenzene derivatives with C15 skeleton, C16 skeleton, and flavonolignans with C6–C3 lignan precursors [88]. Flavonoids are divided into six subclasses; isoflavones, flavones, flavanols, flavonols, flavanones, and anthocyanins, are abundant in plants and their metabolic routes have been thoroughly explored using biochemical and molecular approaches [89,90]. Many plants, including pomelos, blueberries, roselle, oranges, grapefruit, lemons, and limes, are all rich in flavonoids [91].

**Figure 4:**
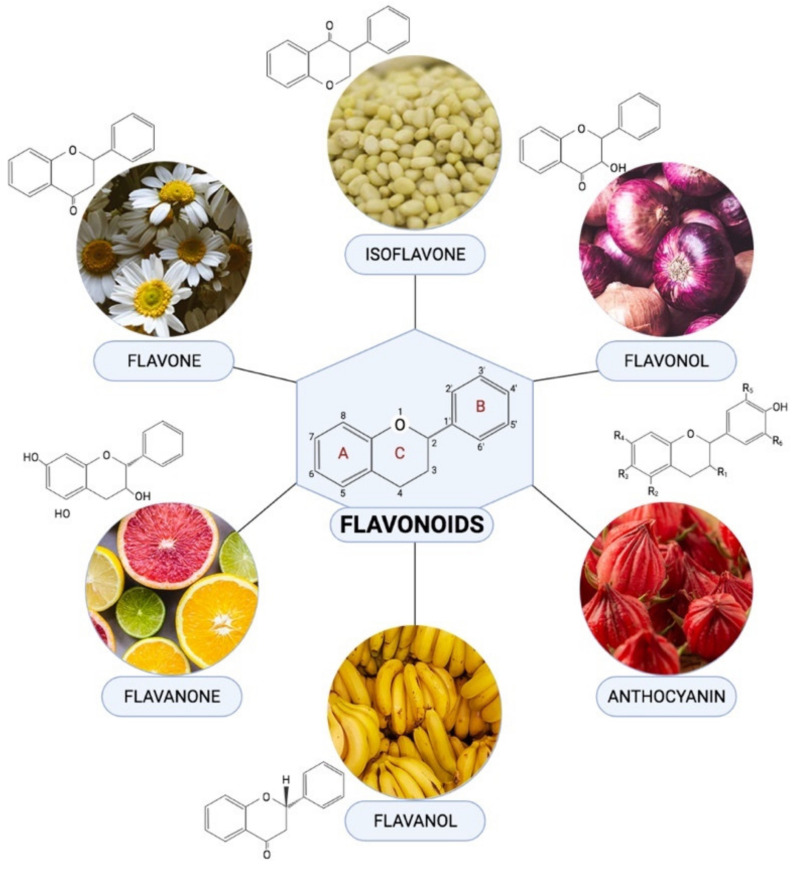
Chemical structures and example of sources where they are found abundant in for each flavonoid subclasses.

Flavonoids have been demonstrated to alleviate pathological disorders, such as diabetes, cancer, obesity, and cardiovascular diseases. Flavonoids are abundant plant-based natural compounds with a good potential for medicinal and biological actions. These compounds showed the ability to exert anti-oxidative, anti-inflammatory, anti-fibrotic, and anti-apoptotic activities as reported previously [92,93,94]. Given the role of mitochondrial-induced oxidative stress in the progression of DCM, this review aims to summarize mechanisms of action of flavonoids in alleviating DCM by targeting mitochondrial-induced oxidative stress. Figure 4 demonstrated the chemical structure of flavonoid subclasses.

### 4.1. Flavones

Flavone is one of the significant flavonoid subclasses. They are found as glycosides in celery, parsley, red peppers, mint, and ginkgo biloba. This group of flavonoids includes luteolin, apigenin, and tangeritin [95]. They have a double bond between positions 2 and 3 of the main C ring, as well as a ketone in position 4. The hydroxyl group at position 5 of the A ring is found in the majority of flavones from vegetables and fruits, but hydroxylation in other parts, most commonly in position 7 of the A ring or 3′ and 4′ of the B ring, varies depending on type of the vegetable or fruit [96].

Luteolin is one of the most prevalent flavones that can be found in variety of vegetables, fruits and herbs such as apple, cabbage, carrot, tea, and celery. A previous study by Yang and colleagues [97] which utilizes streptozotocin-induced diabetic rodents ischemia reperfusion model showed that luteolin treatment, at 100 mg/kg dose, was able to increase cardiac MnSOD and endothelium nitric oxide synthase (eNOS) expression as well as decrease Ca^2+^ induced mPTP opening and ΔΨm.

In addition, myricitrin, a flavone that can be found in abundance in berries and teas, has been proven to suppress high glucose-induced superoxide production in mitochondria, depolarization of mitochondrial membrane potential and restored mPTP formation in diabetic cardiomyopathy via in vitro study [98]. Another study has reported that flavonoid from *Abroma augusta* L. (Malvaceae) leaf extract containing predominantly rutin improved co-enzyme Q_9_ and Q_10_ levels in the mitochondria by acting as antioxidants through scavenging ROS and thereby inhibit lipid peroxidation [99].

### 4.2. Isoflavones

Isoflavones are phytocompounds with a chemical composition based on the 3-phenyl chromen-4-one backbone. Isoflavones are secondary plant metabolites extensively studied for its wide range of therapeutic effects, including antioxidant, chemopreventive, anti-inflammatory, anti-allergic, antibacterial, and cardioprotective effects [100,101]. The highest content of isoflavones is identified to be in roots and seeds. Other medicinal plants with high isoflavones content include red clover, dyer’s broom, lucerne, and sohphlang flax. Beside soy, other legumes such as lupin beans, kudzu, barley, and fava beans are rich in isoflavones [102,103]. The most important types of isoflavones are genistein, daidzein, glycitein, formononetin, biochanin A, and equol [104].

Isoflavone has been reported to alleviate mitochondrial-induced oxidative stress on DCM. Recently, Upadhayay et al. [24] reported that isoflavone was able to reduce mitochondrial-induced oxidative damage by reducing ROS generation in mitochondria and depolarization of mitochondrial membrane through silent information regulator 1 (SIRT-1) pathway or PPAR-α, which further attenuated mitochondrial dysfunction, thus conserving cardiomyocytes health. Besides, recent study conducted by Laddha and colleagues has confirmed that streptozotocin-induced type 1 diabetic rats were shown to maintain AMPK and SIRT-1 levels to normal levels whereby both activities are important in controlling free fatty acid uptake as well biogenesis in cardiac mitochondria [105].

However, studies made on the effects of isoflavones on cardiac diabetes model are rather meagre in number. Yet, we can still refer to its therapeutic effect on other cardiac pathology models as well. There are several studies that reported favorable effects of isoflavones on cardiac mitochondria. Recently, isoflavones was shown to give positive effects on mitochondria by alleviating the excessive mitochondria Ca^2+^ uptake in isolated heart [106]. Apart from that, isoflavones was also capable in improving disturbance in ΔΨm as well as reduction of intracellular ROS release, thus proving that isoflavone was able limit oxidative stress induced by mitochondria [107,108]. In addition, isoflavones also alleviate ΔΨm loss as well as curbing mPTP opening which exhibiting cardiac protective effect [108].

### 4.3. Flavonol

Flavonoids with a ketone group are known as flavonols. Flavonols can be found plentiful fruits and vegetables. Kaempferol, quercetin, myricetin, and fisetin are among the most widely studied flavonols and they can be found in abundant in common daily diet including in onions, kale, lettuce, apples, and berries. Flavonol consumption has been proved to a variety of health advantages, including antioxidant potential and a lower risk of cardiovascular disease. Flavonols have a hydroxyl group in position 3 of the C ring, which can be glycosylated, unlike flavones. Flavonols have a vast spectrum of methylation and hydroxylation forms, and they are the most prevalent and largest subclass of flavonoids in fruits and vegetables based on their many glycosylation patterns [109].

Flavonol has a vast potential in protecting heart mitochondria. Earlier study has revealed that flavonol was able to enhance mitochondrial biogenesis by increasing mitochondrial DNA content via upregulation of nuclear factor erythroid 2-related factor, Nrf-1, Nrf-2, and mitochondrial transcription factor A (TFAM) expression [110]. Furthermore, flavonol also was capable to improve complexes I, III, and IV activities as well as upregulate expression of UCP-2 and UCP-3 [110]. These findings show that flavonols have promising capability in protecting against cardiovascular disease development.

Indeed, previous study reported that flavanols found in *Abroma augusta* L. family of Malvaceae including rutin, myricetin, and quercetin have been proven to revive the ubiquinones (co-enzyme Q) function, which is important in electron carriers’ distribution within cell organelles chiefly and thus reduce ROS production in myocardial mitochondrial of T2DM [99]. In another cardiac study model, quercetin also was found to control free FAO by modulating phosphorylation of AMPK via upregulation of AMPKα2, PPARα, and PCG-1α genes where these genes are crucial in altered energy metabolism mechanisms [111,112]. The derivative of myricetin, dihydromyricetin, could boost mitochondrial performance in streptozotocin-induced diabetic rodents, thus reducing oxidative stress. In this study, the ATP levels and complexes Ι/ΙΙ/ΙΙΙ/ΙV maneuver in ETC as well as ΔΨm were enhanced when treated with dihydromyricetin in the cardiomyocytes [113].

Recently, Ni and colleagues [23] have demonstrated that flavonol icariin could upregulate Apelin, the gene in myocardium and the mitochondrial matrix gene Sirt3, hence elevates the ΔΨm and reduces mitochondria ROS production. Another study has shown that flavonol from quercetin could induce peroxiredoxin-3 (Prx-3) expression, a mitochondrial antioxidant, causing a significant decrease in myocardial biomarkers for mitochondrial uncoupling and redox stress, UCP3 protein expression, and reduction of cardiac thioredoxin-2 (Trx-2) expression as well as thioredoxin reductase-2 (TrxR2) activity. Therefore, it could upregulate the expression of Nrf2/Nrf1 and consequently elevate Prx-3 expression [114]. An in vivo study conducted by using *Murraya koenigii* (curry) and *Moringa oleifera* leaf extract that contain quercetin and kaempferol has reported that these flavonols were able to enhance the expression SOD1 gene, PGC 1α gene, and ATPase and improve mitochondrial function in the diabetic heart [115].

The disruption of mitochondrial transmembrane potential is one of the causes that lead to mitochondrial induce oxidative stress. Taxifolin (dihydroquercetin), a subclass of flavonol could restore mitochondrial transmembrane potential in H9c2 cell lines (Sun et al. 2014). Wu and the team [113] have reported that the dihydromyricetin could enhance the ATP content levels, citrate synthase activity and complex Ι/ΙΙ/ΙΙΙ/ΙV and ATP synthase activities as well as increase in ΔΨm.

### 4.4. Flavanol

Flavanols are the 3-hydroxy derivatives of flavanones commonly known as dihydroflavonols or catechins. They are a multi-substituted and highly diverse subclass of flavonoids. [96]. Due to the hydroxyl group attached to position 3 of the C rings, flavanols are also known as flavan-3-ols. There is no double bond between positions 2 and 3, unlike many flavonoids. Fruits such as bananas, pears, apples, blueberries, and peaches are rich in flavanols.

In earlier study, flavanol has appear to protect heart mitochondria via various mechanisms. This includes protection effect of flavanol via meddling with ETC complexes activities through deprivation of complex I activity, consequently mitochondrial membrane depolarization which then of ROS production (NO and H_2_O_2_) [116]. This has been corroborated by previous study on T2DM model where epigallocatechin-3-gallate (EGCG), a flavanol, attenuated myocardial deterioration and showed beneficial effects on myocardial mitochondrial components. EGCG has been demonstrated to revive complex I, III, and IV, as well as voltage-dependent anion-selective channel 1 (VDAC1) activities that produce major ROS. Mitochondrial DNA (mtDNA) copies and the mitochondrial dehydrogenase activity were significantly revived in treatment model [117]. This evidence indicated that EGCG could be an effective substances to protect mitochondria-induced oxidative stress in cardiomyocytes of T2DM.

Moreover, epicatechin is one of the flavanols reported to attenuate DCM through modulation of mitochondrial-induced oxidative stress. Ramírez-Sánchez and colleagues [118] have demonstrated that epicatechin could block the suppressive effect of high glucose on heart mitochondrial biogenesis involving mitofilin, SIRT1, PGC-1α, and TFAM levels. Treatment with epicatechin also has reversed the high level of eNOS-O-GlcNAc in the diabetic heart.

### 4.5. Anthocyanins

In terrestrial plants, anthocyanins are one of the most common and abundantly distributed secondary metabolites. Anthocyanins are responsible for red, purple, and blue colors in the flowers, seeds, and fruits of numerous plant species. [119,120]. Anthocyanins are natural antioxidants because they are electron deficient, making them highly reactive to ROS [121]. More than 600 anthocyanins have been extracted from a variety of plant species. They are based on the flavylium ion, which has a single fundamental core structure. As a result, the C15 skeleton is formed with a chromane ring with a second aromatic ring B in position 2 (C6-C3-C6) containing single or more sugar molecules attached at various hydroxylated sites on the basic structure. The C3 hydroxyl in the C ring commonly conjugates sugar molecules to the anthocyanidin structure [121]. Anthocyanins and anthocyanin-rich foods have been found to exhibit a variety of biological functions mostly as an antioxidant that may benefit for human wellbeing [122]. The role of anthocyanins has been proven to improve DCM through modulation of mitochondrial-induced oxidative stress. Anthocyanins mainly can be found in plant such as roselle, blackberries and blackcurrants [123]. However, the study that was conducted by using anthocyanins in targeting mitochondrial-induced oxidative stress in DCM is still very limited.

Mitochondria damage is a key factor leading to cardiomyocytes impairment and cell death as well as other cardiac diseases and making mitochondria an attractive target for pharmacological interventions. As a matter of fact, protocatechuic acid (PCA), a primary metabolite of anthocyanins that found in roselle, has been shown to possess as an antioxidant. In an in vitro study by Semaming et al. [122], PCA significantly reduced mitochondrial ROS level and attenuated mitochondrial membrane depolarization. They also found that PCA treatment attenuated mitochondrial swelling as the ROS level decrease. Not only that, PCA treatment alone was able to reduce blood glucose level via enhancing GLUT4 translocation and adiponectin secretion caused by elevated PPARɣ activity in adipocytes. This shows that mediating this mechanism is crucial in alleviating increase of FAO in the mitochondria [124].

Although the use of anthocyanins on diabetic cardiomyopathy research has not yet been extensively investigated, we can presume the result of its interventions by looking at findings of its impact on different cardiac disease models. Previously, anthocyanins has proven to attenuate oxidative stress by scavenge ROS via various mechanisms including direct scavenge ROS, induction of enzymes (superoxide dismutase, catalase) responsible for ROS removal or modulation of ROS forming enzymes (NADPH oxidase) in mitochondria [125,126]. This has been confirmed by another study reporting that anthocyanins was able to quench ROS and thus preserve mitochondrial complex activities in heart [127].

### 4.6. Flavanones

Flavanones are another important compound found in citrus fruits, including oranges, lemons, and grapes. This group of flavonoids includes hesperidin, naringenin, and eriodyctiol. Because of their free radical-scavenging characteristics, flavanones have been linked to various health advantages [96]. Citrus fruit juice and peel contain these substances, which give them a bitter taste. Citrus flavonoids have pharmacological actions that include antioxidant, anti-inflammatory, anti-hyperglycemia, and anti-hypercholestrolemia. The C ring is saturated in flavanones (saturated double bond between positions 2 and 3), giving them the alternative name of dihydroflavonols, and distinguishes them from flavones [109].

Naringin, a major flavanone glycoside found mostly in citrus fruits, has been reported to alleviate mitochondrial-induce oxidative stress by preventing the high glucose-induced loss in mitochondrial membrane potential [128]. In another study conducted by You and colleagues [129], naringin also reduced the downregulation of mitochondrial ATP-sensitive potassium channels, which is important in sensing the metabolic changes in pancreatic beta cells and thus protecting the cardiomyocytes against the hyperglycemic condition.

**Figure 5:**
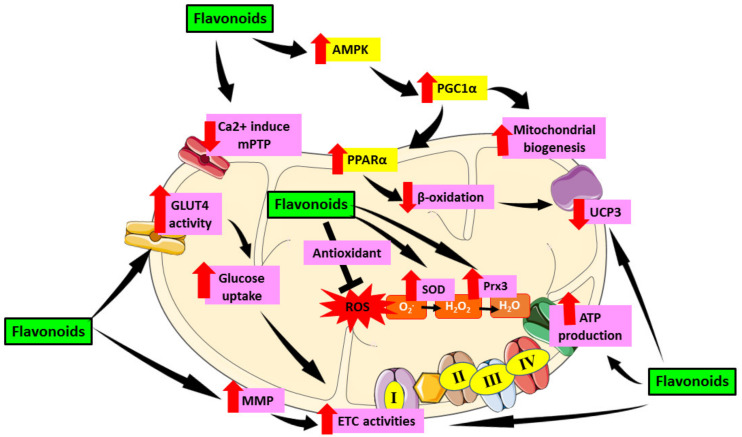
Role of flavonoids in targeting mitochondrial induce oxidative stress in DCM. Flavonoids have been proven to alleviate mitochondrial dysfunction by targeting mechanisms involving oxidative stress in mitochondria including activation of AMPK which then activate PGC1α. PGC1α enhances mitochondrial biogenesis as well as PPARα expression and cause reduction of β-oxidation in mitochondria which then downregulate UCP3. Flavonoids also reduce the formation of Ca^2+^ induced mPTP which preserve mitochondrial number and prevent apoptosis. Furthermore, flavonoids were proven to enhance GLUT4 activity which then led to increase glucose uptake as well as enhance MMP that cause increase in ETC activities which further elevate ATP production. Moreover, flavonoids can act as antioxidant and scavenge ROS as well as increase SOD and Prx3 enzyme which later attenuate oxidative stress. This figure is illustrated based on the review of the previous research. Black arrow indicates flow of mechanisms; red arrow indicates increase/decrease of level/activity.

Similar to anthocyanins, the extensive study on the effect of flavanone in mitochondrial-induced oxidative stress in DCM is still limited. However, in other cardiac models, flavanone has been demonstrated to modulate mitochondrial function in cardiomyocytes. Previously, flavanone was found to ameliorate mitochondrial disruption in cardiomyocytes by reducing impaired mitochondrial membrane potential and suppressing mitochondrial ROS levels and increase mitochondrial antioxidant via regulation of AMPK-mTOR signaling pathways [130,131]. Moreover, flavanone was able to alleviate mitochondrial membrane potential collapse and preserve mitochondrial complex II activity on isolated heart mitochondria [131]. Aside from that, Ca^2+^ overload was reduced significantly with the treatment of flavanone and hence reviving mitochondrial function in the heart [132]. Table 1 shows an overview of the role of flavonoids in alleviating mitochondrial-induced oxidative stress in DCM. Figure 5 demonstrated the role of flavonoids in targeting mitochondrial induce oxidative stress in DCM.

## 5. Future Prospects of Flavonoids Aiming at Reducing Mitochondrial-Induced Oxidative Stress in Diabetic Cardiomyopathy

Mitochondrial dysfunction is the hallmark of cardiac degeneration in DCM. Therefore, it is of utmost importance to curb and alleviate mitochondrial dysfunction in patients with DM. Given enormous evidence linking that mitochondrial-induced oxidative damage can lead to progression of DCM, it is reasonable to assume that lessening oxidative damage would protect the cardiac against detrimental adjustment by diabetes. To the best of our knowledge, there is no clinical study using flavonoids as an antioxidant targeting mitochondrial-induced oxidative stress in DCM. In addition, the preclinical study in this field also is limited. To sustain the improvement of a specific and successful therapy, future studies should investigate the use of flavonoid-rich source targeting specifically in mitochondrial ROS production pathways. Nevertheless, the inhibition of mitochondrial ROS supply might be a useful strategy to prevent the alteration caused by oxidative stress on myocardial structure and function.

We postulate that the lack of clinical trial on this topic could be due to several reasons. This could be a consequence of difficulties in getting human cardiac mitochondrial samples as it is invasive or a lack of proven biomarkers indicative of mitochondrial ROS production, specifically from DCM. Taking into consideration of previous findings obtained in DCM experimental models with the treatment of flavonoids, it is worth assessing whether the flavonoid compound could be used for the treatment of patients with DCM caused by mitochondrial-induced oxidative stress. Flavonoids can be considered as a tool to prevent DCM in patients with DM or to be use in the conjunction with existing treatment to lower the morbidity and mortality rate due to DCM.

## 6. Conclusions

In a nutshell, it is clear that prolonged hyperglycemia can cause mitochondrial-induced oxidative stress and lead to development of DCM. There are scientific evidence showing that flavonoids can protect cardiomyocytes against mitochondrial-induced oxidative stress caused by DM against perturbations, such as altered energy metabolism, impaired calcium handling, altered mitochondrial biogenesis, and altered ETC. Therefore, flavonoids show promising potential in alleviating DCM by protection against mitochondrial-induced oxidative stress. However, studies exploring this potential are rather scarce especially by using isoflavones, anthocyanins, and flavanones. Several mechanisms were also poorly investigated by previous studies even though it is very important in alleviating mitochondrial-induced oxidative stress and intervene by flavonoid. To prove that flavonoids could suppress mitochondrial-induced oxidative stress that causes DCM, in-depth studies are very much needed in the future especially by looking into its effects on altered ETC, mitochondrial biogenesis and calcium handling. Currently, most studies of the effects of flavonoids on cardiac mitochondrial-induced oxidative stress are focusing on animals and cell culture studies rather than clinical study. Hence, more clinical studies examining the beneficial effect of flavonoids on mitochondrial-induced oxidative stress should be conducted as flavonoid has huge potential in ameliorate DCM which then reduce heart failure risk in diabetic patients.

## Figures and Tables

**Table 1:** Summary of flavonoid and its subclasses in targeting mitochondrial-induce oxidative stress in DCM.

Flavonoid Subclass	Type	Study Design	Dose	Results	Reference
Anthocyanin	Protocatechuic acid	In vivo; T1DM Sprague-Dawley rats	50 and 100 mg/kg/day	Reduce mitochondrial ROS levels, attenuated mitochondrial depolarization and decreased mitochondrial swelling in cardiomyocytes.	[122]
Flavones	Vitexin	In vitro; H9C2 cells	1, 3, 10, and 30 µM	Improve mitochondrial ATP production Revive mitochondrial respiratory function by increasing expression of levels of COX IV and SDHB in H9c2 cells.	[25]
	Rutin	In vivo; T2DM Wistar rats	100 and 200 mg/kg/day	Improve co-enzyme Q9 and Q10 in the mitochondria.	[99]
	Luteolin	In vivo; T1DM Sprague-Dawley rats	100 mg/kg/day	Increase MnSOD and eNOS expression and decrease Ca^2+^ induced mPTP opening and mitochondrial inner membrane in cardiomyocytes.	[97]
Isoflavones		In vitro; H9C2 cells	20–200 μg/mL	Reduce mitochondrial-induce oxidative by lowering mitochondrial ROS generation, depolarization of ΔΨm through SIRT-1 pathway or PPAR-α which further attenuate mitochondrial dysfunction and thus conserve cardiomyocytes health.	[24]
		In vivo; T1DM Sprague-Dawley rats	25, 50, and 100 mg/kg orally	Maintained the AMPK and SIRT1 levels.	[105]
Flavonol	Icariin	In vivo and in vitro; db/db, db/+ mice and C57 mice cardiomyocytes	7.5, 15, and 30 µM	Upregulate myocardium gene apelin and the cardiac mitochondrial matrix gene Sirt3. Increase the mitochondrial membrane potential. Reduce mitochondria ROS production.	[23]
Flavonol	Quercetin	In vivo and in vitro; T1DM Wistar rats and H9C2 cells	50 mg/kg and 1 and 10 μM	Induce Prx-3 expression, causing downregulation in myocardial UCP3 protein. Reduce cardiac Trx-2 expression and TrxR2 activity. Induce the expression of transcription factor Nrf2/Nrf1.	[114]
	Quercetin and Kaempferol	In vivo; T1DM albino rats	200 mg/kg/twice daily	Enhance the expression SOD1 gene, PGC 1α gene and ATpase and improve mitochondrial function.	[115]
	Taxifolin/dihydroquercetin	In vivo and in vitro; T1DM C57BL/6 mice and H9C2 cells	10, 20, and 40 µg/mL and 25, 50, and 100mg/kg/day	Restore mitochondrial transmembrane potential in H9c2 cell lines.	[133]
	Dihydromyricetin	In vivo; T1DM C57BL/6 mice	100 mg/kg/day	Enhance ATP levels, CS activity, and complex Ι/ΙΙ/ΙΙΙ/ΙV activities, increase ΔΨm.	[113]
Flavanol	Epigallocatechin-3-gallate	In vivo; T2DM Goto–Kakizaki rats	100 mg/kg/day	Revive Complex I, III, IV, and VDAC1 activities as well as recover mtDNA copies and the mitochondrial dehydrogenase activities.	[117]
	Epicatechin	In vivo and in vitro; T2DM C57BL/6 mice and HCAEC cells	100 nM and 1 mg/kg/day	Blocked the suppressive effect of high glucose on heart mitochondrial biogenesis involving mitofilin, SIRT1, PGC-1α, TFAM protein levels and reversed the high level of eNOS-O-GlcNAc of diabetic heart.	[118]
Flavanone	Naringin	In vitro; H9C2 cells	5 μM	Prevent the HG-induced loss in ΔΨm.	[128]
	Naringin	In vivo and in vitro; T1DM Sprague-Dawley rats and H9C2 cells	80 μM and 25, 50, and 100 mg/kg/day	Reduce the downregulation of KATP channels.	[129]

## Data Availability

No new data were created or analyzed in this study. Data sharing is not applicable to this article.

## Abbreviations

DM	Diabetes mellitus
CVD	Cardiovascular disease
DCM	Diabetic cardiomyopathy
ROS	Reactive oxygen species
AGE	Advanced glycation end product
PKC	Protein kinase C
ATP	Adenine triphosphate
OXPHOS	Oxidative phosphorylation
DNA	Deoxyribonucleic acid
mPTP	Mitochondrial permeability transition pore
FAO	Fatty acid oxidation
PPAR	Peroxisome proliferator-activated receptor
AMPK	5′ adenosine monophosphate-activated protein kinase
PGC-1	Peroxisome proliferator-activated receptor-gamma coactivator 1
NADH	Nicotinamide adenine dinucleotide (NAD) + hydrogen (H)
FADH2	Flavin adenine dinucleotide
ETC	Electron transport chain
MnSOD	Manganese superoxide dismutase
UCP	Uncoupling protein
ΔΨm	Inner mitochondrial membrane
MMP	Mitochondrial membrane polarization
O-GlcNAc	O-linked β-N-acetylglucosamine
OGT	O-GlcNAc transferase
NOX	Nicotinamide adenine dinucleotide phosphate oxidase
UCP-DTA	Uncoupling Protein-diphtheria Toxin A
eNOS	Endothelial nitric oxide synthase
SIRT1	Silent information regulator 1
Nrf	Nuclear factor erythroid 2–related factor 2
TFAM	Mitochondrial transcription factor A
Prx3	Peroxiredoxin-3
Trx2	Thioredoxin-2
VDAC	Voltage-dependent anion channel 1
EGCG	Epigallocatechin-3-gallate
PCA	Protocatechuic acid
GLUT4	Glucose transporter type 4
